# The importance of endothelial protection: the emerging role of defibrotide in reversing endothelial injury and its sequelae

**DOI:** 10.1038/s41409-021-01383-x

**Published:** 2021-09-28

**Authors:** Paul G. Richardson, Marta Palomo, Nancy A. Kernan, Gerhard C. Hildebrandt, Nelson Chao, Enric Carreras

**Affiliations:** 1 Jerome Lipper Multiple Myeloma Center, Dana-Farber Cancer Institute, Harvard Medical School, Boston, MA USA; 2 Barcelona Endothelium Team, Josep Carreras Leukaemia Research Institute, Hospital Clinic/University of Barcelona Campus, Barcelona, Spain; 3 Hematopathology, Department of Pathology, Centre de Diagnostic Biomedic (CDB), Hospital Clinic de Barcelona, Institut d’Investigacions Biomediques August Pi i Sunyer (IDIBAPS), Universitat de Barcelona, Barcelona, Spain; 4 Pediatric BMT Service, Memorial Sloan Kettering Cancer Center, New York, NY USA; 5 Markey Cancer Center, University of Kentucky, Lexington, KY USA; 6 Duke Cancer Institute, Duke University Medical Center, Durham, NC USA

**Keywords:** Cancer, Haematological cancer

## Abstract

Hepatic veno-occlusive disease/sinusoidal obstruction syndrome (VOD/SOS), a potentially life-threatening complication of hematopoietic cell transplantation (HCT), results from prolonged sinusoidal endothelial cell activation and profound endothelial cell damage, with sequelae. Defibrotide, the only drug approved in the United States and Europe for treating VOD/SOS post-HCT, has European Commission orphan drug designation for preventing graft-versus-host disease (GvHD), associated with endothelial dysfunction. This endothelial cell protector and stabilizing agent restores thrombo-fibrinolytic balance and preserves endothelial homeostasis through antithrombotic, fibrinolytic, anti-inflammatory, anti-oxidative, and anti-adhesive activity. Defibrotide also preserves endothelial cell structure by inhibiting heparanase activity. Evidence suggests that downregulating p38 mitogen-activated protein kinase (MAPK) and histone deacetylases (HDACs) is key to defibrotide’s endothelial protective effects; phosphatidylinositol 3-kinase/Akt (PI3K/AKT) potentially links defibrotide interaction with the endothelial cell membrane and downstream effects. Despite defibrotide’s being most extensively studied in VOD/SOS, emerging preclinical and clinical data support defibrotide for treating or preventing other conditions driven by endothelial cell activation, dysfunction, and/or damage, such as GvHD, transplant-associated thrombotic microangiopathy, or chimeric antigen receptor T-cell (CAR-T) therapy-associated neurotoxicity, underpinned by cytokine release syndrome and endotheliitis. Further preclinical and clinical studies will explore defibrotide’s potential utility in a broader range of disorders resulting from endothelial cell activation and dysfunction.

## Introduction

Defibrotide, a polydisperse mixture of predominantly single-stranded polydeoxyribonucleotide sodium salts, was identified in the 1960s in the search for potential treatments for coagulation and thrombotic disorders that would have a lower risk of hemorrhage than other anticoagulants. Originally derived from bovine lung, defibrotide is now produced via controlled depolymerization of porcine intestinal DNA [1–3]. Defibrotide has demonstrated antithrombotic, fibrinolytic, anti-inflammatory, anti-oxidative, and anti-adhesive activity, but has shown no systemic anticoagulant effects, with no effects on coagulation parameters such as partial thromboplastin or prothrombin times [1, 4].

Defibrotide was first approved for clinical use in Italy in 1986 to treat deep vein thrombosis, an indication expanded in 1993 to include treatment and prevention of vascular disease with risk of thrombosis [5]. By the mid-1990s, defibrotide was considered for treating severe veno-occlusive disease/sinusoidal obstruction syndrome (VOD/SOS) as part of a study searching for potential novel therapies targeting endothelial injury; its positive impact on endothelial stress was confirmed with correlative studies in the same setting [6, 7]. As of 2020, defibrotide is the only drug approved for treating hepatic VOD/SOS. In the United States, defibrotide is indicated for treatment of adult or pediatric patients with hepatic VOD/SOS with renal or pulmonary dysfunction following hematopoietic cell transplantation (HCT) [2]. In Europe, defibrotide is indicated for treatment of severe hepatic VOD/SOS following HCT in patients aged >1 month [8]. In addition, the European Commission granted orphan drug designation to defibrotide in 2013 for prevention of graft-versus-host disease (GvHD) [9].

VOD/SOS, a potentially life-threatening HCT complication, is caused by prolonged sinusoidal endothelial cell activation and subsequent injury from radiation or toxic metabolites of chemotherapy within HCT conditioning regimens [10–12]. Endothelial cell damage increases expression of von Willebrand factor (vWF), which stimulates platelet aggregation, and tissue factor, which promotes activation of coagulation factors. Endothelial cell damage also increases expression of inflammatory mediators, such as intercellular adhesion molecule-1 (ICAM-1) and tumor necrosis factor-α (TNF-α), and releases heparanase, which results in loss of cytoskeletal structure [13–16]. In the liver, as the sinusoidal endothelium deteriorates, gaps form in the endothelial lining, allowing cellular debris to enter the space of Disse, leading to sinusoidal narrowing. This is accompanied by downstream embolism of sinusoidal endothelial cells which cause a progressive blockage of sinusoidal flow [1, 11, 15, 17]. Changes induced by endothelial cell activation induce a shift in phenotype, from antithrombotic to procoagulant, with hypofibrinolysis reflected by downregulation of tissue plasminogen activator and markedly increased plasminogen activator inhibitor-1 (PAI-1) levels [18], leukocyte adhesion molecule expression, cytokine production, and loss of vascular integrity. These changes appear to be a common pathogenic mechanism underlying a range of conditions [19]. Notably, endothelial cells are not all the same, performing different functions depending on their location in the body. The endothelium arises from cell differentiation in the mesoderm; however, other cell lineages may also transdifferentiate into endothelial cells. Therefore, features such as Weibel–Palade bodies or fenestrae are not found in every endothelial cell. Consequently, endothelial cell heterogeneity has posed a challenge for developing treatments [20].

This article discusses and reviews the proposed mechanism of action of defibrotide, an endothelial cell protector.

### Pharmacological characteristics of defibrotide

Defibrotide is a mixture of 90% single-stranded phosphodiester oligonucleotides and 10% double-stranded phosphodiester oligonucleotides. Within this mixture, various aptamers have been identified with biological actions in vitro, including thrombin inhibition and cathepsin G inhibition [3, 21]. The polyanionic nature of the single-stranded DNA aptamers interferes with cationic proteins that cause vascular instability [22]. These single-stranded DNA aptamers then modulate the function of cationic proteins secreted by activated neutrophils in the context of neutrophil extracellular traps [22, 23]. However, defibrotide degrades to several different products in vivo, so the identity of the clinically active derivative is unclear [4].

A lack of immunogenicity was demonstrated in animal models after prolonged defibrotide administration. A preclinical study using rats and dogs found that treatment with varying doses of defibrotide for a 3-month period did not result in the production of anti-defibrotide, anti-heparin platelet factor 4 (PF4) or antiphospholipid antibodies. This is a notable observation as it contrasts with polyelectrolytes such as heparin which are capable of interacting with endogenous proteins, leading to the formation of antibodies such as anti-heparin PF4 [24].

Defibrotide has a relatively short half-life. In pharmacokinetic studies of healthy volunteers, the mean (standard deviation) half-life was 0.71 (0.35) h after one 6.25-mg/kg dose of defibrotide [8]. A Japanese study reported a mean (standard deviation) half-life of 0.47 (0.10) h after defibrotide 6.25 mg/kg administration to healthy volunteers as a 2-h infusion [25]. Given this relatively short half-life, defibrotide must be administered every 6 h; it can also clear rapidly in case of adverse events [8].

### Cellular effects of defibrotide

**Fig. 1:**
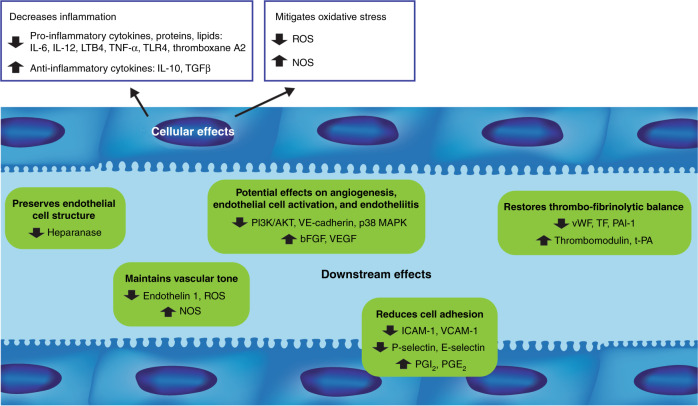
Proposed cellular and downstream effects of defibrotide. IL interleukin, LTB4 leukotriene B4, TNF-α tumor necrosis factor-α, TLR4 Toll-like receptor 4, TGFβ transforming growth factor-β, ROS reactive oxygen species, NOS nitric oxide synthase, PI3K/AKT phosphatidylinositol 3-kinase/Akt, VE-cadherin vascular endothelial cadherin, MAPK mitogen-activated protein kinase, bFGF basic fibroblast growth factor, VEGF vascular endothelial growth factor, vWF von Willebrand factor, TF tissue factor, PAI-1 plasminogen activator inhibitor-1, t-PA tissue plasminogen activator, ICAM-1 intercellular adhesion molecule, VCAM-1 vascular cell adhesion molecule-1, PGI_2_ prostaglandin I2, PGE_2_ prostaglandin E2.

Defibrotide protects endothelial cells via modulation of a wide, complex range of molecular pathways (Fig. 1 and Table 1). In a human endothelial cell line, defibrotide attached to the external cell membrane and then became internalized via macropinocytosis in a concentration-, temperature-, and time-dependent fashion [26]. However, the interaction between defibrotide and the endothelial cell membrane, without internalization, was sufficient to induce anti-inflammatory and antioxidant responses [26]. Defibrotide anti-inflammatory effects include reduced release of inflammatory mediators such as interleukin 6 (IL-6), thromboxane A2, leukotriene B4, and TNF [3], while antioxidant effects include attenuation of reactive oxygen species (ROS) generation and restoration of endothelial nitric oxide synthase levels during oxidative stress in vitro [26]. Interaction with adenosine receptors on the endothelial cell membrane had been considered a critical step in defibrotide’s mechanism of action [27]. In a study in murine dendritic cells, defibrotide-induced production of interleukin 10 was completely inhibited by incubation with the adenosine receptor antagonist 8-p-sulfophenyltheophylline, suggesting that defibrotide acted as an agonist at the adenosine receptor [28]. However, in human endothelial cell line experiments, blocking adenosine receptors with 8-p-sulfophenyltheophylline had no effect on defibrotide attaching to cells and no specific receptor for defibrotide was identified [26].

**Table 1:** Proposed cellular effects of defibrotide.

Cellular effects	Preclinical and clinical evidence
Anti-inflammatory effects	• Reduces release of inflammatory mediators (IL-6, thromboxane A2, LTB4, TNF) [3]
Antioxidant effects	• Attenuates ROS generation and restore endothelial NOS levels during oxidative stress [26]
Protective effects	• Downregulates HDACs [29] • Inhibits the expression of ROS, ICAM-1, vWF, TLR4, VCAM-1, and TF to levels consistent with normal endothelial function [29] • Inhibits the activation of PI3K/AKT and p38 MAPK, as well as a major effector p70S6K, in endothelial cells [26, 33, 36]

*IL* interleukin, *LTB4* leukotriene B4, *TNF* tumor necrosis factor, *ROS* reactive oxygen species, *NOS* nitric oxide synthase, *HDAC* histone deacetylases, *ICAM* intercellular adhesion molecule, *vWF* von Willebrand factor, *TLR* toll-like receptor, *VCAM* vascular cell adhesion molecule, *TF* tissue factor, *PI3K/AKT* phosphatidylinositol 3-kinase/Akt, *MAPK* mitogen-activated protein kinase.

Histone deacetylase (HDAC) downregulation is thought to be a key mechanism of defibrotide’s endothelial protective effects [29]. HDACs are important epigenetic factors regulating pro-inflammatory gene expression in tissues [30, 31]. In endothelial cells, HDACs appear to be involved in modulating inflammation and proliferation in response to shear stresses [30]. Western blot and immunofluorescence techniques showed that defibrotide normalized overexpression of HDAC1 in a dose-dependent manner in human umbilical cord vein endothelial cells exposed to sera of patients with chronic kidney disease, a condition associated with endothelial dysfunction [29]. Furthermore, in endothelial cells exposed to chronic kidney disease patients’ sera, defibrotide inhibited expression of ROS, ICAM-1, vWF, and Toll-like receptor 4 to levels consistent with normal endothelial function [29]. Previous studies showed that HDAC expression is regulated by the phosphatidylinositol 3-kinase/Akt (PI3K/AKT) pathway, as demonstrated by a reduction in shear-stress-induced expression of HDACs by PI3K inhibitors [30, 32]. Several early HCT-associated complications seem to have a more microvascular (i.e., small blood vessel) than macrovascular (i.e., large blood vessel) location.

Defibrotide has inhibited PI3K/AKT activation in endothelial cells derived from human umbilical vein and an immortalized human microvascular cell line induced by HCT patients’ sera [33] and in microvascular endothelial cells with AKT activation stimulated by exposure to GvHD patient serum [34]. In addition, defibrotide blocked activation of p70S6K, a major PI3K/AKT pathway effector, in microvascular endothelial cells [35]. Furthermore, defibrotide prevented increased HDAC1 and HDAC2 expression in endothelial cell cultures incubated with P740-Y-P, a PI3K/AKT pathway activator [29]. Thus, it appears that one mechanism of defibrotide’s endothelial protective effects reduced HDAC upregulation through PI3K/AKT pathway inhibition, making PI3K/AKT the potential link between defibrotide’s interaction with the endothelial cell membrane and its downstream effects. Moreover, defibrotide has decreased activation of p38 mitogen-activated protein kinase (MAPK), which occurs even in the context of micropinocytosis inhibition. This suggests that defibrotide may act at the endothelial cell membrane to alter intracellular p38 MAPK signaling [26]. Supporting this as a potential key construct to defibrotide’s mechanism of action, p38 MAPK pathway upregulation activates pro-inflammatory cytokines, such as IL-6, TNF-α, and IL-1β, thus contributing to numerous inflammatory pathologies, including endotheliitis [36]. In summary, p38 MAPK pathway downregulation may be an important additional mechanism of defibrotide’s anti-inflammatory and endothelial protective cellular effects.

### Downstream effects of defibrotide

Defibrotide acts on multiple pathways that affect endothelial homeostasis (Fig. 1 and Table 2). Defibrotide protects endothelial cells and reduces their activation and subsequent dysfunction via antithrombotic, fibrinolytic, anti-inflammatory, anti-oxidative, anti-adhesive, anti-apoptotic, and anti-angiogenic mechanisms, thereby restoring thrombotic-fibrinolytic balance and preserving endothelial homeostasis [1, 3, 4, 33, 37–41].

**Table 2:** Proposed downstream effects of defibrotide.

Downstream effects	Preclinical and clinical evidence
Effects on thrombo-fibrinolytic balance	• Inhibits thrombin-induced platelet aggregation, thromboxane biosynthesis, and fibrin clot formation [21] • Prevents increased expression of vWF [33, 34] • Reduces reactivity of the endothelial cell matrix toward platelets induced by exposure to GvHD serum [34] • Reduces LPS-induced tissue factor expression, tissue factor procoagulant activity, and tissue factor antigen in microvascular endothelial cells [40] • Stimulates thrombomodulin expression in macrovascular endothelial cells [42] • Enhances the activity of plasmin to degrade fibrin clots formed by fibrinogen, plasminogen, and thrombin [39] • Prevents LPS-induced increase in PAI-1 expression and enhanced LPS-induced increase in t-PA antigen expression, resulting in a net increase in fibrinolytic activation [40]
Reduction of cell adhesion	• Inhibits leukocyte adhesion by interfering with LFA-1/ICAM-mediated leukocyte transmigration in vitro [45] and by suppressing P-selectin in rats [46] • Stimulates the production of PGI_2_ and PGE_2_ [38] • Suppresses acute GvHD serum-induced expression of VCAM-1, ICAM-1, and vascular-endothelial cadherin [34] • Inhibits mononuclear cells and CD3+ T cells while downregulating expression of the adhesion molecules E-selectin, P-selectin, VCAM-1, and ICAM-1 [48] • Blocks the increased expression of ICAM-1 in patients undergoing autologous HCT [33]
Anti-inflammatory/antioxidant properties	• Decreases the release of inflammatory mediators (IL-6, thromboxane A2, LTB4, TNF, and ROS) [3, 26] • Decreases levels of the pro-inflammatory cytokines (IFNγ, TNF-α, IL-6, and IL-12) [48] • Increases levels of the anti-inflammatory mediators (TGFβ and IL-10) [48] • Maintains vascular tone by antagonizing the vasoconstrictor activity of endothelin 1 and enhancing the production of NO and NOS [3]
Endothelial cell protection and anti-apoptotic effects	• Protects endothelial and epithelial cells from F-Ara–induced apoptosis [41] • Inhibit the activity and cell surface expression of heparanase, VEGF, ICAM-1, and E-selectin in multiple myeloma and mesothelioma cells [49]
Anti-angiogenic effects	• Binds and mobilizes bFGF, while protecting it from oxidative and protease degradation and potentiating its binding to FGFR1-IIIc [53] • Inhibits formation of new blood vessels and reduces tumor microvascular density [35] • Reduces acute GvHD-induced expression of VE-cadherin and suppresses endothelial cell proliferation and endothelial cell tube formation [34]

*vWF* von Willebrand factor, *HCT* hematopoietic cell transplantation, *GvHD* graft-versus-host disease, *LPS* lipopolysaccharide, *PAI-1* plasminogen activator inhibitor-1, *t-PA* tissue plasminogen activator, *LFA-1* lymphocyte function-associated antigen-1, *ICAM* intercellular adhesion molecule, *PGI*_*2*_ prostaglandin I2, *PGE*_*2*_ prostaglandin E2, *VCAM* vascular cell adhesion molecule, *IL* interleukin, *LTB4* leukotriene B4, *TNF* tumor necrosis factor, *ROS* reactive oxygen species, *IFNγ* interferon-γ, *TGFβ* transforming growth factor-β, *NO* nitric oxide, *NOS* nitric oxide synthase, *VEGF* vascular endothelial growth factor, *bFGF* basic fibroblast growth factor, *FGFR* fibroblast growth factor receptor.

#### Effects on thrombo-fibrinolytic balance

Several in vitro studies have demonstrated that defibrotide modulates antithrombotic and fibrinolytic factors. Three aptamers from defibrotide were shown to be potent inhibitors of thrombin-induced platelet aggregation, thromboxane biosynthesis, and fibrin clot formation in vitro [21]. In human umbilical vein endothelial cells (macrovascular) and microvascular endothelial cells, defibrotide prevented increased expression of vWF induced by exposure to sera from autologous HCT recipients or newly diagnosed GvHD patients [33, 34]. Defibrotide also reduced the increased endothelial cell matrix reactivity toward platelets induced by GvHD serum exposure [34]. In microvascular endothelial cells, defibrotide reduced lipopolysaccharide-induced tissue factor expression, tissue factor procoagulant activity, and tissue factor antigen [40]; in macrovascular cells, incubation with defibrotide stimulated thrombomodulin expression [42].

Fibrinolytic properties were demonstrated in an in vitro experiment in which defibrotide bound to human plasmin and enhanced plasmin activity to degrade fibrin clots formed by fibrinogen, plasminogen, and thrombin [39]. In macro- and microvascular endothelial cells, defibrotide prevented lipopolysaccharide-induced increase in PAI-1 expression and enhanced lipopolysaccharide-induced increase in tissue plasminogen activator antigen expression, resulting in a net increase in fibrinolytic activation with defibrotide [40]. Human study findings mirror these in vitro results. In healthy volunteers, a single defibrotide dose counteracted a natural circadian fall in tissue plasminogen activator and accelerated the PAI-1 level decrease seen in placebo-treated participants, suggesting defibrotide acts to stimulate circulating fibrinolytic activity [38]. In another healthy volunteer study, a single defibrotide dose induced an increased level of systemic tissue factor pathway inhibitor, which plateaued between 5 and 20 min post-dose and returned to basal levels by 40 min post-dose, a profile similar to that following a single dose of low-molecular weight heparins, enoxaparin, and nadroparin [37]. In patients with VOD/SOS, defibrotide decreased PAI-1 and increased tissue plasminogen activator levels, increasing overall fibrinolytic activity [43].

Although defibrotide has been shown to reduce platelet adhesion and aggregation, it has not shown significant systemic anticoagulant effects or effects on coagulation parameters, such as partial and activated thromboplastin times and prothrombin time [4, 44]. An in vitro study using human blood products showed that defibrotide increased plasmin activity with no direct effect on plasminogen activation to plasmin, suggesting a local fibrinolytic potential for defibrotide [39].

#### Reduction of cell adhesion

Defibrotide has demonstrated anti-adhesive properties in vitro and in preclinical and human studies. In cell cultures, defibrotide inhibited leukocyte adhesion to endothelial cells under basal conditions and after endothelial cell stimulation. Defibrotide inhibited leukocyte adhesion by interfering with lymphocyte function-associated antigen-1/intercellular adhesion molecule-mediated leukocyte transmigration in vitro [45] and by suppressing P-selectin in rats [46]. In platelet-perfused guinea pig hearts, defibrotide administration resulted in selective stimulation of prostaglandin I2 but had no effect on release of prostaglandin E2 or thromboxane [47]. However, in human volunteers, defibrotide stimulated production of prostaglandin I2 and prostaglandin E2, likely through stimulating the arachidonate pathway in leukocytes or by platelet-leukocyte interactions [38].

In vitro studies also examined defibrotide’s effects on GvHD patient serum-induced changes in adhesion parameters. In microvascular endothelial cell cultures, defibrotide significantly suppressed acute GvHD serum-induced expression of vascular cell adhesion molecule-1, ICAM-1, and vascular-endothelial cadherin [34]. In macrovascular endothelial cell cultures exposed to GvHD sera followed by defibrotide, trans-endothelial migration of mononuclear cells and CD3+ T cells was inhibited, along with a downregulated expression of adhesion molecules E-selectin, P-selectin, vascular cell adhesion molecule-1, and ICAM-1 compared with cultures not treated with defibrotide [48]. Results were similar in macrovascular and microvascular endothelial cells exposed to sera from autologous HCT patients, in which defibrotide blocked increased expression of ICAM-1 [33]. These findings suggest that defibrotide’s protective actions in the endothelium include downregulating expression of endothelial adhesion molecules overexpressed in response to GvHD or HCT.

#### Anti-inflammatory and antioxidant properties

In endothelial cells, defibrotide exerts anti-inflammatory and antioxidant properties by decreasing release of inflammatory mediators such as IL-6, thromboxane A2, leukotriene B4, TNF, and ROS [3, 26]. In a mouse model of GvHD, mice with ongoing acute GvHD post-HCT had increased levels of pro-inflammatory cytokines interferon-γ, TNF-α, IL-6, and interleukin 12, with reduced levels of anti-inflammatory mediators transforming growth factor-β and interleukin 10, compared with control mice. Prophylactic defibrotide treatment produced a marked decrease in pro-inflammatory mediators and an increase in anti-inflammatory mediators compared with untreated mice [48]. Defibrotide also maintained vascular tone by antagonizing endothelin 1 vasoconstrictor activity and enhancing production of nitric oxide and nitric oxide synthase. These effects are potentiated by reduction of ROS formation, which in turn improves the bioavailability and effectiveness of endothelium-derived nitric oxide [3].

#### Endothelial cell protection and anti-apoptotic effects

The pathways through which defibrotide protects against chemotherapy-related endothelial damage do not appear to impact chemotherapy-induced antitumor activity. In microvascular endothelial cell culture, apoptosis was induced by incubation with F-Ara, the active metabolite of fludarabine; defibrotide pretreatment or coadministration protected endothelial cells from F-Ara–induced apoptosis [41]. Similar defibrotide protective effects were observed for epithelial cells from keratinocyte and alveolar cell lines, a potentially relevant observation given that skin is the most frequent site of acute GvHD manifestation and the lung significantly contributes to transplant-related mortality in idiopathic pneumonia syndrome and diffuse alveolar hemorrhage [41]. Another study looked at in vitro effects of defibrotide combined with a series of antitumor agents, including chemotherapies and biologic agents, on multiple myeloma and endothelial cells [49]. Defibrotide had no effect on these agents’ antitumor activity and no antitumor activity of its own. However, in the same study, defibrotide inhibited heparanase activity in multiple myeloma cells and, while adding exogenous heparanase increased the invasion potential of multiple myeloma cells, defibrotide treatment abolished this effect [49]. Furthermore, the interaction of multiple myeloma cells with microvascular endothelial cells increased expression of heparanase, vascular endothelial growth factor, ICAM-1, and E-selectin in endothelial cells; defibrotide suppressed these effects [49]. Another study also found that defibrotide inhibited heparanase activity and tumor growth in mesothelioma cells [50]. Heparan sulfate proteoglycans are crucial in maintaining the structure and stability of the extracellular matrix and basement membrane [51], so inhibiting heparanase expression may contribute to defibrotide’s protective effects on the endothelium. Interestingly, high levels of heparanase in recipients compared with donors have been associated with acute and chronic GvHD following HCT, suggesting a role for heparanase in hyperactivation of donor T cells toward recipient tissues and a potential rationale for heparanase-targeting treatments [52].

#### Anti-angiogenic effects

Defibrotide effects on angiogenic mechanisms appear to be complex. In vitro, defibrotide has been shown to bind with high affinity to basic fibroblast growth factor (bFGF) and mobilize bFGF from its binding sites on the endothelial extracellular matrix, while protecting bFGF from oxidative and protease degradation and potentiating its binding to fibroblast growth factor receptor [53]. As bFGF is known to promote formation of microvessels and stimulate expression of vascular endothelial growth factor, these results suggest that defibrotide’s action in VOD/SOS may be partly related to its ability to promote partial revascularization of injured hepatic tissue, through direct bFGF enhancement and indirect effects on vascular endothelial growth factor-induced angiogenesis [53]. However, evidence also suggests that defibrotide has anti-angiogenic activity. As discussed previously, defibrotide inhibits PI3K/AKT, an important signaling pathway for angiogenesis [35]. In vitro and in vivo data suggest that defibrotide anti-angiogenic effects occur, not by disrupting tumor endothelium, but by inhibiting formation of new blood vessels and reducing tumor microvascular density [35]. Similarly, in a mouse model of mesothelioma, defibrotide treatment inhibited tumor growth associated with impaired tumor vascular density [50]. In endothelial cell culture, defibrotide reduced acute GvHD-induced expression of vascular endothelial-cadherin, an adherence junction protein involved in angiogenesis and vascular integrity; defibrotide also suppressed endothelial cell proliferation and endothelial cell tube formation [34].

### Predictive biomarkers for defibrotide

Identification of potential biomarkers for defibrotide efficacy has proven complex given its pleiotropic effects. Kaleelrahman et al. studied the potential role of PAI-1 levels in diagnosing hepatic VOD/SOS post-HCT and response to subsequent defibrotide treatment. In a study involving five patients with suspected VOD/SOS post-HCT, PAI-1 levels decreased after defibrotide treatment in the four patients showing VOD/SOS resolution, but remained elevated in the patient who did not respond to defibrotide [54]. In an exploratory analysis of a phase 2 dose-finding study of defibrotide in 151 patients with severe VOD/SOS post-HCT, PAI-1 plasma concentrations were lower (although this difference was not statistically significant) at Days 7 and 14 of defibrotide treatment compared with baseline in patients with complete response and in patients who survived beyond Day 100 [55]. Decreased or stable bilirubin has also been associated with better response to defibrotide [55], suggesting that early bilirubin and/or PAI-1 changes could be potential pharmacodynamic markers for efficacy, as could reduced serum creatinine based upon results of an earlier study, along with improved platelet count [7].

### Clinical implications

Given its role in promoting endothelial homeostasis, defibrotide has therapeutic potential for conditions driven by endothelial dysfunction. Based on its identified pharmacologic actions, defibrotide has been evaluated in disorders involving endothelial cell activation, endotheliitis, and/or endothelial damage.

#### VOD/SOS

Thus far, defibrotide has been most extensively studied for treating and preventing VOD/SOS post-HCT. Defibrotide is currently the only drug approved for treatment of adult and pediatric patients with hepatic VOD/SOS following HCT [2, 8]. In a phase 3 open-label, historically controlled study in 102 patients with VOD/SOS with multi-organ failure, Day 100 survival post-HCT was 38% with defibrotide versus 25% in the historical control group (*P* = 0.0109), with complete response rates of 26% versus 13%, respectively (*P* = 0.0160) [56]. These findings were replicated in a larger open-label, expanded-access study of defibrotide in 1000 patients with VOD/SOS with or without multi-organ dysfunction following HCT (Day 100 survival: 59%). Among patients with multi-organ dysfunction (*n* = 512), Day 100 survival was 50% [57]. Defibrotide also demonstrated efficacy for prevention of VOD/SOS in a phase 3, open-label, randomized trial of 356 high-risk pediatric patients undergoing HCT; prophylactic defibrotide reduced VOD/SOS incidence at 30 days post-HCT versus controls (12% vs 20%; *P* = 0.0488) [58]. An ongoing phase 3 trial of defibrotide for VOD/SOS prophylaxis (NCT02851407) recently stopped enrollment after meeting protocol-defined criteria for futility, suggesting a low probability of meeting the primary endpoint of demonstrating a significant 30-day VOD/SOS-free survival difference with the sample size estimates and specific methodology used; analyses are ongoing and results are not yet reported, but no safety concerns were described [59]. Overall, there were no significant differences in hemorrhage rates with defibrotide versus controls in published studies, supporting the safety of this therapeutic approach in such critically ill patients [56, 58]. Early intervention has also been consistently associated with better outcomes in adult and pediatric populations, and published prevention studies show a benefit in high-risk populations with favorable tolerability [58].

#### GvHD

Based on its mechanism of action, defibrotide is also being studied for GvHD prevention. In a murine model of GvHD, the survival rate was 88% in the defibrotide-treated group while the untreated group had no survivors; defibrotide benefits were also observed after the occurrence of GvHD [48]. An exploratory analysis of the phase 3 pediatric VOD/SOS prevention trial showed that patients who received defibrotide prophylaxis had a lower incidence and severity of acute GvHD versus the control group [58]. A study of defibrotide prophylaxis in adults receiving HCT (*N* = 195) also suggested a role for defibrotide in decreasing incidence and severity of acute GvHD: incidence of acute GvHD was 26% for patients who received defibrotide pre-HCT, 40% for those receiving defibrotide post-HCT, and 47% for those receiving no defibrotide (*P* = 0.057), with a trend toward a lower rate of severe GvHD in the pre-HCT arm versus the other groups (*P* = 0.051) [60]. A phase 2 open-label trial evaluating defibrotide for prevention of acute GvHD after HCT in children and adults completed in May 2020 (NCT03339297) [61].

Defibrotide has demonstrated protective effects on endothelial cells from HCT conditioning and data suggest some efficacy in the prevention of acute GvHD after HCT [48, 61]. The endothelial cells that cover the vascular tree are directly exposed to damaging factors that occur during the onset and progression of acute GvHD [34]. Previous findings suggested that acute GvHD occurrence is associated with activation and dysfunction of endothelial cells [34, 48]. These results suggest that the endothelium may be a potential target for GvHD prevention and treatment therapies, of which defibrotide may play an important role in improving endothelial tolerance as part of the GvHD pathobiology.

#### Transplant-associated thrombotic microangiopathy (TA-TMA)

TA-TMA, another common complication following HCT, is associated with widespread endothelial dysfunction across many organs, providing the rationale for the potential therapeutic use of defibrotide for this condition [62]. In a retrospective study of defibrotide in 17 adults and 22 pediatric patients, TA-TMA resolved in 77% of patients. Notably, patients with TA-TMA resolution were generally diagnosed earlier than those without TA-TMA resolution, suggesting that earlier identification and defibrotide treatment may improve outcomes [62]. Investigator-initiated studies for this indication are in progress.

#### Chimeric antigen receptor T-cell (CAR-T) therapy-associated neurotoxicity

Defibrotide may have potential utility in treating endothelial cell injury associated with cellular therapy, for example, CAR-T therapy-associated neurotoxicity. Patients receiving CAR-T therapy often develop cytokine release syndrome, a systemic inflammatory response, which may require intensive care with respiratory support and can lead to neurotoxicity with symptoms including confusion, hallucinations, and seizure [30, 63]. A study of adults with severe CAR-T therapy-associated neurotoxicity found clinical evidence of endothelial dysfunction, including vascular instability, capillary leak, disseminated intravascular coagulation, and blood–brain barrier disruption, along with elevated biomarkers of endothelial activation and endotheliitis, including vWF, IL-6, and TNF-α [64]. Endothelial stabilizing agents with anti-inflammatory properties, like defibrotide, may help prevent severe neurotoxicity, warranting further study based on defibrotide’s demonstrated mechanism of action. An ongoing phase 2 trial is evaluating efficacy and safety of defibrotide for prevention of CAR-T therapy-associated neurotoxicity in patients with relapsed or refractory diffuse large B-cell lymphoma receiving axicabtagene ciloleucel, with preliminary results expected in 2021 (NCT03954106) [65].

#### Cerebral malaria

Disruption of the blood–brain barrier is known to contribute to manifestations of cerebral malaria, including intracerebral hemorrhage, seizures, and increased intracranial pressure [66]. Multiple mechanisms may underlie blood–brain barrier endothelial cell dysfunction, such as adhesion of parasitized red blood cells to endothelium, inflammatory cytokine response, and blood coagulation, all offering possibilities for therapeutic intervention [66]. A preclinical study using a murine model of cerebral malaria investigated defibrotide effects on aspects of malaria pathogenesis [28]. In vitro, defibrotide blocked tissue factor expression and reduced prothrombinase activity, platelet aggregation, and complement activation, demonstrating its antithrombotic activity along with its ability to be anti-inflammatory and reverse endothelial injury. In *Plasmodium falciparum* culture, adding defibrotide prevented parasite growth, possibly by inhibiting red blood cell invasion by released merozoites [28]. This preclinical evidence suggests that the effects of defibrotide coadministration with anti-malarial drugs may warrant further study.

#### Sickle-cell disease

Sickle-cell disease is often characterized by painful vaso-occlusive crisis, with underlying mechanisms thought to be multifactorial, involving inflammation, adhesion, and aggregation of sickled red blood cells with endothelial cells and platelets [67]. A preventive treatment targeting these underlying mechanisms could aid in management of the condition and potentially reduce reliance on powerful analgesics and their associated complications [67]. To that end, defibrotide is under investigation in a phase 2 trial (NCT03805581) in patients with sickle-cell disease-associated acute chest syndrome, with results anticipated by 2022 [68].

#### COVID-19 pneumonia

COVID-19, a disease caused by a new coronavirus, is associated with severe lung complications, such as pneumonia and acute respiratory distress syndrome, but vascular complications predominate, including a unique endotheliitis and associated microangiopathy. Data from murine models of acute lung injury and idiopathic pneumonia syndrome suggest that defibrotide protects against endothelial cell injury and stabilizes endothelial cell integrity in this setting [69]. Researchers are studying the potential role of defibrotide in reducing progression of acute respiratory failure rate in COVID-19 pneumonia, and specifically reversing the endotheliitis that underpins the syndrome and leads to profound endothelial cell dysfunction in advanced disease. Pilot clinical studies and phase 2 trials are underway, with promising early results in both U.S. and E.U. studies [70–73].

## Conclusions

A large body of both preclinical and clinical literature supports a mechanism of action for defibrotide that reduces endothelial cell activation and damage, thereby restoring the thrombo-fibrinolytic balance and preserving endothelial homeostasis. The totality of evidence suggests that defibrotide exerts endothelial protective effects through downregulating HDACs, with PI3K/AKT and p38 MAPK as the potential intracellular link between defibrotide interaction with the endothelial cell membrane and its downstream effects.

Potential limitations associated with defibrotide treatment include frequent infusions placing a high demand on patients and requiring an inpatient setting, although efforts to produce a subcutaneous formulation are underway. Treatment cost may also impact patient access and utilization; however, results from a Canadian study showed that defibrotide treatment for VOD/SOS with multi-organ dysfunction was cost-effective, with an incremental cost-effectiveness estimate below the current payer threshold [74]. While the clinical utility of defibrotide in treating VOD/SOS has been established, its role in preventing VOD/SOS or treating other diseases that result from endothelial dysfunction, such as GvHD, is under investigation. Ongoing and future clinical studies will provide insights into the therapeutic potential of defibrotide for preventing or treating other conditions driven by endothelial cell activation, endotheliitis, and endothelial dysfunction. In summary, defibrotide is a unique, naturally derived endothelial cell protector that restores thrombo-fibrinolytic balance and preserves endothelial homeostasis through antithrombotic, fibrinolytic, anti-inflammatory, anti-oxidative, and anti-adhesion activities. It has proven remarkably safe with broad potential in an array of critical disease states underpinned by endothelial cell damage and inflammation. As such, it constitutes a novel, first-in-class oligonucleotide in a new category of promising biopharmaceuticals, which modulate arguably one of the most important interfaces in human disease, namely the endothelium.
